# Patient experience and challenges in group concept mapping for clinical research

**DOI:** 10.1186/s41687-019-0147-9

**Published:** 2019-08-15

**Authors:** Geoffrey D. Mills, Marianna LaNoue, Alexzandra T. Gentsch, Amanda M. B. Doty, Amy Cunningham, Garrison Nord, Kristin L. Rising

**Affiliations:** 1 Department of Family and Community Medicine, Thomas Jefferson University, 833 Chestnut Street, Suite 301, Philadelphia, PA 19107 USA; 2 College of Population Health, Thomas Jefferson University, Philadelphia, PA USA; 3 Department of Emergency Medicine, Thomas Jefferson University, Philadelphia, PA USA

**Keywords:** Group concept mapping, Patient reported outcomes, Diabetes, Quality of life, Brainstorming

## Abstract

**Background and objective:**

Group concept mapping (GCM) is a research method that engages stakeholders in generating, structuring and representing ideas around a specific topic or question. GCM has been used with patients to answer questions related to health and disease but little is known about the patient experience as a participant in the process. This paper explores the patient experience participating in GCM as assessed with direct observation and surveys of participants.

**Methods:**

This is a secondary analysis performed within a larger study in which 3 GCM iterations were performed to engage patients in identifying patient-important outcomes for diabetes care. Researchers tracked the frequency and type of assistance required by each participant to complete the sorting and rating steps of GCM. In addition, a 17-question patient experience survey was administered over the telephone to the participants after they had completed the GCM process. Survey questions asked about the personal impact of participating in GCM and the ease of various steps of the GCM process.

**Results:**

Researchers helped patients 92 times during the 3 GCM iterations, most commonly to address software and computer literacy issues, but also with the sorting phase itself. Of the 52 GCM participants, 40 completed the post-GCM survey. Respondents averaged 56 years of age, were 50% female and had an average hemoglobin A1c of 9.1%. Ninety-two percent (*n* = 37) of respondents felt that they had contributed something important to this research project and 90% (*n* = 36) agreed or strongly agreed that their efforts would help others with diabetes. Respondents reported that the brainstorming session was less difficult when compared with sorting and rating of statements.

**Discussion:**

Our results suggest that patients find value in participating in GCM. Patients reported less comfort with the sorting step of GCM when compared with brainstorming, an observation that correlates with our observations from the GCM sessions. Researchers should consider using paper sorting methods and objective measures of sorting quality when using GCM in patient-engaged research to improve the patient experience and concept map quality.

## Background

Group Concept Mapping (GCM) is a relatively complex and labor-intensive mixed-methods approach for stakeholder engagement in identifying and organizing ideas around a particular topic [1]. The approach takes place over multiples steps. The first step involves having stakeholders work together to brainstorm responses to a statement (“focus prompt”). In the second step, participants use GCM software [2] to independently sort all of the brainstormed ideas into piles based on similarity. During this step, participants may also be asked to rate each of the brainstormed ideas based on predetermined criteria such as importance or feasibility. After participants have completed this step, the software uses a statistical technique known as multidimensional scaling to aggregate all of the participants’ sorting and create a concept map. On the concept map, each brainstormed idea is represented by a point on the map, with the distance between any two points on the map representing their relatedness based on the participant sorting data (i.e. closer together = sorted together by more participants). The software also suggests groupings of the points into more general clusters based on the similarities of the points. In the final GCM stage, participants are brought back together as a group to review the concept maps, refine the cluster boundaries to ensure that the group agrees with how the ideas have been grouped together, and name the final clusters to be representative of the ideas contained within the clusters [1, 3].

GCM has been traditionally applied in education [4], theory development [5], program evaluation and intervention planning [6] but has potential to engage patients in answering health services and biomedical research questions [7, 8]. In this context, GCM may efficiently engage patients in setting research priorities and better understanding the patient experience of health and disease [9]. We used group concept mapping (GCM) to engage with patients living with diabetes mellitus (DM) to understand their priorities when seeking treatment for DM [9]. During the GCM sessions, we observed that our patient-participants had varying challenges throughout the process, and many were unable to complete the sorting and rating phase (step 2) without assistance from a research team member.

Given the centrality of participant engagement throughout the GCM process, limitations in patients’ ability to complete specific GCM tasks are potential barriers to meaningful engagement of patients in GCM. However, few studies have elicited participant feedback on the GCM experience or acknowledged participant challenges as a process limitation. In this work, we sought to describe specific challenges of patients in completing the GCM process and to gain a better understanding of the overall patient experience participating in GCM. We hope to inform whether and how to most effectively include patients as participants in future GCM research.

## Methods

This study is part of a larger study that was conducted to compare the methods of individual interviews and GCM for engaging patients to identify patient-important outcomes related to seeking care [9]. For this study, we report findings from direct research team observations of participant involvement during the GCM sessions, as well as results from a telephone-administered patient survey administered after completion of GCM activities. The entire study was conducted in close collaboration with our Patient Advocate and Key Stakeholders Advisory Board (PAKSAB). The study received institutional IRB approval [9, 10].

### Study setting and participants

We recruited adult, English-speaking patients with a history of type 1 or 2 DM from a large urban academic healthcare system in Philadelphia (USA). Patients were eligible to participate if, within the past 3 months, they 1) had presented to the emergency department (ED) with a DM-related problem (acute), 2) were admitted to the hospital for a DM-related problem (post-acute), or 3) had a primary care visit and had 2 or more HbA1c labs > 7.5% in the prior year (primary care). Patients were excluded if they had a new diagnosis of DM, had significant complications related to DM (end stage renal disease, amputation, or blindness), were undergoing medical clearance for a detox center, were in police custody, had major communication barriers (visual or hearing impairment or dementia), or were determined unable to provide informed consent.

We convened three distinct GCM iterations, each of which included a mix of patients representing each of the three care settings. Participants were recruited from these three distinct periods on the care continuum to allow for capturing any potential variation in patient goals and preferences related to current health status. Patients were identified from the electronic medical record and were contacted by telephone in a random order for screening to assess interest in participation. We recruited for a target participation of 16–20 patients in each GCM iteration. Final participation ranged from 14 to 24 in each group. Written informed consent was obtained prior to the start of any GCM activities.

Each GCM iteration was scheduled to begin on a Saturday and had three in-person sessions: brainstorming, sorting and rating, and idea refinement. We collected demographic and patient-reported clinical information with a survey before initiation of GCM activities. Brainstorming occurred in the morning and lasted approximately 90 min. We used the following prompt, developed by our PAKSAB, for brainstorming: “You’re here as a person with diabetes; when people with diabetes seek care, what are they hoping to improve or make happen?” Participants were invited to write down responses for several minutes, then shared ideas verbally. The research team recorded all responses on a list at the front of the room; participants were encouraged to continue providing responses until the group felt that no new ideas were emerging. Similar ideas were then combined to produce the final list of patient-important outcomes.

The sorting and rating tasks occurred after a lunch break and took between 45 and 140 min. Upon returning from lunch, each participant sat at a computer with the brainstormed statements loaded into Concept Systems Global Software [2]. The research team provided an overview of the sorting and rating tasks and a demonstration of the software. Participants were instructed to individually sort the brainstormed statements into piles based on perceived similarity by dragging and dropping statements into piles in the software. Once the statements were sorted, they named each of their piles. For the rating activity, participants each read and rated every brainstormed statement on importance and achievability: “How important is this goal to you personally when thinking about your diabetes care?” (1 = “not at all important” to 5 = “very important”) and “How achievable is this goal for you personally?” (1 = “not at all achievable” to 5 = “very achievable”). The final session (idea refinement) took place the following Monday evening. In this session, participants reviewed and refined the draft concept maps and named the final clusters as a group. This session took two to two-and-a-half hours. Concept mapping participants were compensated $125 for completing all three sessions associated with a single GCM iteration.

To assess participant challenges and experiences with GCM, we recorded the research team’s real-time observations during the sorting and rating phase of GCM (step 2). Sorting and rating is the only part of the process that is done individually, and thus the only time that input from and computer use by each participant is required. We tracked the frequency of requests to the research team for assistance from the participants and the types of assistance provided. Assistance requests were categorized as brief questions regarding software use, conceptual difficulties with the sorting and/or rating, computer literacy (difficulty with using the software, keyboard, or mouse), visual impairment, and literacy challenges. Brief questions were resolved quickly with research staff (less than 1–2 min) and extended assistance was recorded when participants required help from staff for most or all of the sorting and rating phases.

To gain patient perspectives regarding their experiences and challenges participating in GCM, we also developed a 17-question survey (referred to in the following as the “engagement survey”) that included questions about overall patient experience with GCM, comfort with various steps in the process, and suggestions for improvement. The survey format employed 16 questions with defined Likert scales, two of which had open-ended follow-up questions. Additionally, the final survey question was open-ended (“Is there anything else you think would be helpful for us to know?”). Whenever possible, Likert-scale questions used balanced wording (e.g., “How easy or hard was it to …”). For all Likert items, equal numbers of negative and positive response options were provided to avoid bias. After the last GCM session, we administered the survey to GCM participants via telephone and, as a result, the time between GCM participation and survey completion ranged from 1.5 weeks to 3 months. We analyzed patient responses to 13 questions specifically related to the patient experience with GCM. The survey study received approval from the Thomas Jefferson University Institutional Review Board.

### Data analysis

Descriptive statistics were calculated for survey participants (and non-participants) characteristics including ranges for relevant variables. Categorical counts of research team interactions with GCM participants were reported with a breakdown of types of assistance provided.

## Results

Fifty-two patients participated in one of the three GCM iterations (GCM-A, GCM-B, and GCM-C), and 40 (76%) completed the engagement survey. Eight participants could not be reached, three declined to participate, and one participant who was highly disruptive during a GCM session was not contacted. The characteristics of the respondents and non-respondents are shown in Table 1. All 11 non-respondents (100%) identified as non-Hispanic black, compared to 31 (78%) of respondents. A majority of respondents (24, 61%) reported an income of less than $24,999. Respondents had an average hemoglobin A1c of 9.1%.

**Table 1:** Characteristics of the Survey Respondents and Non-Respondents

	Participated (*n* = 40) *n* (%)	Declined/Could Not Be Reached (*n* = 11) *n* (%)
Age, years -mean (range)	56 (23–76)	55 (24–94)
Gender		
Male	20 (50)	5 (45)
Female	19 (48)	6 (55)
Declined to answer	1 (2)	
Ethnicity		
Hispanic or Latino	3 (7)	0 (0)
Not Hispanic or Latino	37 (93)	11 (100)
Race		
Black	31 (78)	11 (100)
Caucasian/White	5 (13)	0 (0)
Asian	1 (2)	0 (0)
Some other race	2 (5)	0 (0)
Declined to answer	1 (2)	0 (0)
Education		
Less than high school	8 (20)	0 (0)
High school/GED/some college	20 (50)	9 (82)
College degree	10 (25)	2 (18)
Post graduate degree	2 (5)	0 (0)
Income		
< $10,000	3 (8)	4 (36)
$10,000 - $24,999	21 (53)	1 (9)
$25,000 - $49,999	10 (25)	4 (36)
$50,000 - $99,999	1 (2)	2 (18)
$100,000+	2 (5)	
Data wasn’t collected	2 (5)	
Declined to answer	1 (2)	
Hemoglobin A1C	*n* = 35	*n* = 10
mean, (range)	9.1 (5.3–14.8)	10 (7–14.7)
Data not recorded	5 (13)	1 (9)
Initial Recruitment Setting		
Emergency Department	15 (37.5)	4 (36.4)
Ambulatory	15 (37.5)	4 (36.4)
Post-Hospitalization	10 (25)	3 (27.2)
GCM Session		
A (*n* = 24)	18 (75)	6 (25)
B (n-13)	11 (85)	2 (15)
C (*n* = 14)	11 (79)	3 (21)

### Frequency of research team interactions with patient participants

Individual members of the research team reported the number of times participants asked for their assistance and what type of assistance they gave each participant (Table 2). There were a total of 92 assistance requests, with 33 (36%) extended assists from research staff and 59 (64%) brief assists. The most common type of assistance needed was help using the computer during the sorting and rating phases of GCM (60, (65%)) though many patients required additional conceptual assistance with sorting. Two patients (2%) had visual impairments that limited their ability to independently use the computer, and two other patients (2%) had literacy issues that required extended assistance from the research team to complete the sorting and rating tasks. Additionally, two patients (2%) left during the sorting phase without completing the task.

**Table 2:** Research Team Interactions with GCM Participants

	GCM-A	GCM-B	GCM-C	Total
Total reported brief assists	221	213	165	59
Total reported extended assists	18	11	4	33
Type of assistance^a^				
Sorting concept issues	14	6	8	28
Brief software questions	6	16	9	31
Computer literacy issues	17	10	2	29
Visual impairment	1	1	0	2
Literacy issues	1	1	0	2
Participants left before completincompleting sorting	1	0	1	2

^a^ Some individuals required multiple brief assists

### Patient engagement and personal value of GCM

**Fig. 1:**
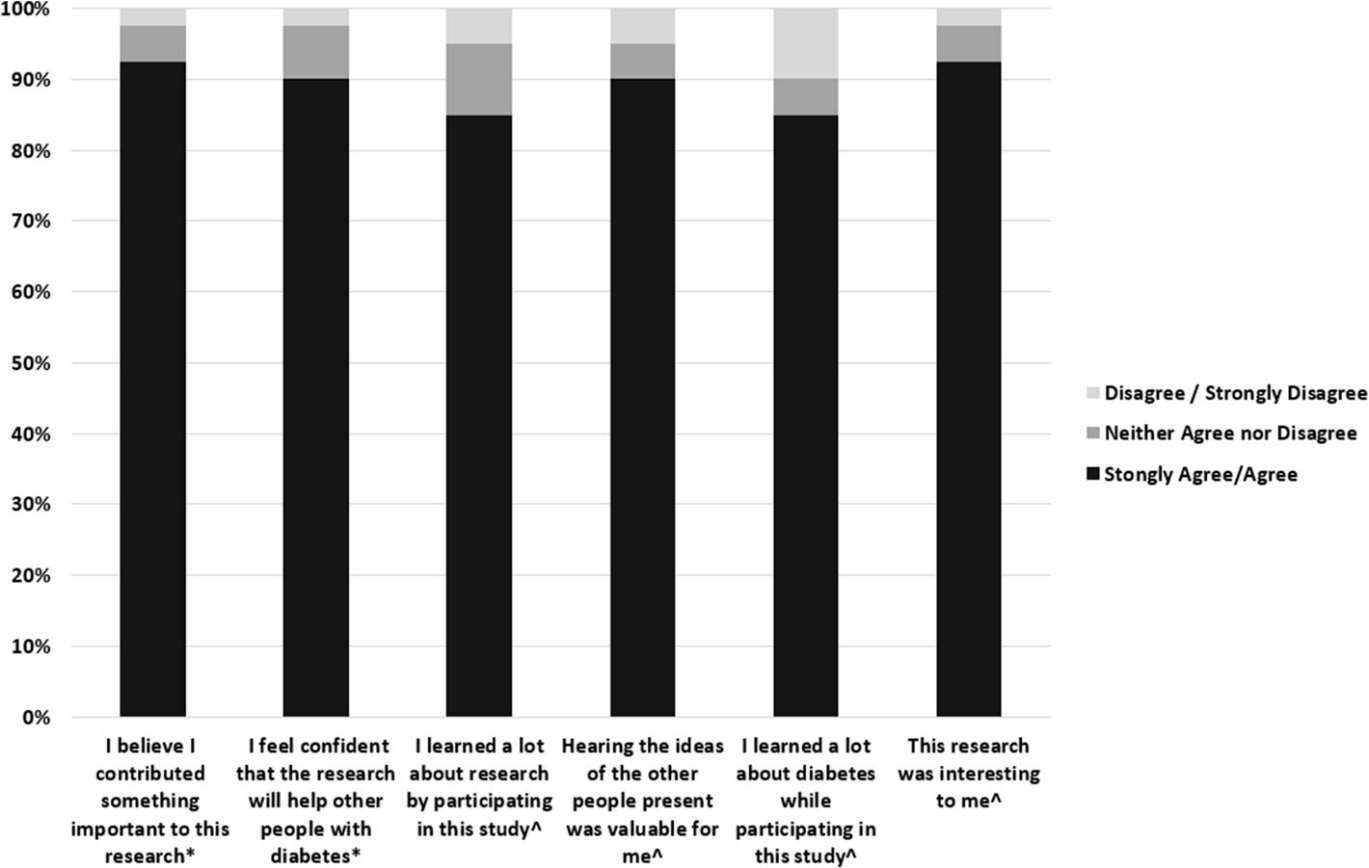
Patient Engagement* and Personal Value^ Measures

Figure 1 shows survey responses to the 13 items related to patient engagement and personal value of GCM. Patients felt that they had contributed something important to this research project (37, 93% agree/strongly agree) and most patients agreed or strongly agreed that our research would help others with diabetes (36, 90%). We asked patients about the personal value of participation. Many patients reported that hearing ideas of other participants was valuable (36, 90% agree/strongly agree) and less reported learning about diabetes (34, 85% agree/strongly agree). The mean score across the 6 participant-experience questions was 4.43 (SD = 0.57) (Likert-scale 1 = strongly disagree to 5 = strongly agree). Patients were asked for subjective comments about their experience. One participant said, “I just learned something from it, I got a whole lot out of it”. Another said, “Some things I didn’t really understand as the day went on.”

### Patient comfort with GCM steps

Table 3 shows survey responses about the GCM process. Most felt that they had participated sufficiently: (26, 65% strongly disagreed or disagreed with the statement “I didn’t participate as much as I wanted to”) and reported that group brainstorming was easy or very easy (29, 73%). Because of our observation that patients struggled with sorting and rating, we asked about the patient experience specific tasks. Most respondents reported being comfortable using the computer for the sorting and rating (30, 75% comfortable/very comfortable). Under half (19, 48%) of the participants found sorting easy or very easy, and 6 (15%) respondents reported that the sorting step was hard or very hard. Regarding the final step of GCM (refinement), most respondents (24, 62%) reported that it was easy or very easy to decide which cluster each item belonged in during the final review of the clustered statements, yet 18 (46%) did not find naming easy or very easy. Patient comments regarding their experience with the computers includied the following: “Hard part was computers,” “Computer part was difficult,” and “It was so many questions with the computer, and some of it was easy, but it was lengthy.”

**Table 3:** Process Measures

	*n* (%)				
	Strongly agree	Agree	Neither agree nor disagree	Disagree	Strongly disagree
I didn’t participate as much as I wanted to.	2 (5)	6 (15)	6 (15)	9 (22.5)	17 (42.5)
Participating in this research study was frustrating.	0	1 (2.5)	4 (10)	10 (25)	25 (62.5)
	Very comfortable	Comfortable	Neutral	Uncomfortable	Very uncomfortable
How comfortable did you feel using a computer?	24 (60)	6 (15)	5 (12.5)	2 (5)	3 (7.5)
	Very easy	Easy	Neutral	Hard	Very hard
How easy or hard was it to answer the research question during the brainstorming session?	20 (50)	9 (22.5)	10 (25)	1 (2.5)	0
How easy or hard was it to decide which pile each statement should go into (sorting)?	11 (27.5)	8 (20)	15 (37.5)	5 (12.5)	1 (2.5)
How easy or hard was it to decide as a group if a statement needed to be moved to another pile?	13 (32.5)	11 (27.5)	10 (25)	3 (7.5)	2 (5)
How easy or hard was it to name each (sorted) pile?	11 (27.5)	10 (25)	13 (32.53)	5 (12.5)	0

## Discussion

Among our population of patients with poorly-controlled DM, we found that many patients required hands-on assistance from the research team to complete the sorting and rating tasks of GCM due to both cognitive and physical limitations. Despite challenges completing the activities, however, most patients valued their participation in GCM, with some even reporting having learned about diabetes from others in the group through the process.

Our observations and survey findings about challenges with the sorting phase of GCM is reflected in other GCM studies [8, 11]. One study using GCM to understand barriers to cancer screening in formerly homeless women with serious mental illness excluded 11 of 27 sorts (41%) in their analysis due to sorting statements based on importance, using a “miscellaneous” pile, or only sorting into two piles [8]. Another study about quality of life in residential long-term care showed that patients had a significantly harder time with both sorting and rating when compared to staff and family participants [12]. A pooled study analysis of GCM showed that completion rate for sorting and rating tasks were 74% for face-to-face GCM application and only 52% for web-based systems [13]. Though participant challenges were reported in these studies, the challenges were not explicitly discussed as process limitations for GCM. In a study of family members of adults with intellectual disabilities and professionals with experience working with this patient population, 9 of 56 sorting phase participants (16%) reported that the step was difficult and time consuming [14]. Another study exploring mental health disorders in 20 Javanese patients reported challenges when some participants placed a majority of statements into individual piles resulting in clustering errors [11]. In this study, the research team chose to remove 5 (25%) of the patients’ sorts to create usable maps. Finally, in another study of prostate cancer treatment, 6 of 89 sorts (7%) were excluded from the final analysis due to sorts of 3 or fewer piles [15]. Given the centrality of this phase in GCM, this is a potential barrier to GCM use in patient-engaged research, and highlights a need to identify possible solutions.

GCM is a useful tool for patient engagement because patients can complete all data generation and analysis phases with minimal researcher bias [16]. In the larger study we performed from which this analysis arose, we demonstrated that GCM brainstorming was more comprehensive and efficient than interviews for identifying patient priorities related to DM care, and suggest that GCM may be a useful method to elicit other patient-important outcomes [9]. Our findings in this study raise important issues for consideration of when GCM is an appropriate method for patient engagement. Particularly for patients with physical (eyesight, physical control of mouse) or cognitive (understanding what it means for ideas to be conceptually similar) challenges, their ability to engage in GCM may be limited or may require extensive assistance from team members.

While the identified limitations might suggest that GCM is not an ideal method for patient engagement, the patient survey suggests that most patients perceived value in their participation and had a positive experience with the process despite their challenges. We suggest that, with appropriate planning, many of these barriers can be either avoided or overcome. To avoid participation barriers, researchers may consider screening potential participants for literacy and basic computer skills. If screening is not possible or is likely to affect study results (e.g. patients with lower cognitive function are important part of the population for engagement), researchers should be prepared to have extra staff available to provide individual assistance specifically during the sorting and rating step. In addition, researchers may consider providing participants who have challenges using the computer with printed idea statements to use for the sorting phase. Finally, researchers should establish clear a priori criteria during the study design phase for how to determine “adequacy” of each sort for inclusion in analysis.

### Limitations

Although our data draw from a representative sample of patients who have poorly controlled diabetes that accessed our health system, the study is limited by a small sample size that may affect generalizability. Eligible patients self-selected to participate in GCM and then to participate in the post-GCM survey, thus limiting the sample to potentially a more readily engaged and outspoken population. All survey non-responders were black; although the majority of our participants were black (31,78%), this demographic difference in non-responders requires further exploration, as it does not appear to be associated with income or education levels. Every effort was made to choose neutral stems for the post-GCM survey questions but prompt wording may have introduced a positive or negative bias in the responses. Additionally, survey responses may have been influenced by social desirability bias: e.g., participants may have wanted to respond that they felt comfortable with GCM processes to present themselves in a favorable way. Finally, the data gathered about research team assistance with the sorting and rating steps tracked instances of assistance rather than help needed per patient, limiting our ability to determine the proportions of patients who required help.

## Conclusions

While many patients required assistance with completion of various GCM activities, most reported contributing important information and having a positive experience with GCM. With proper consideration and preparation for potential patient challenges to GCM participation, GCM can be a powerful and efficient tool for patient engagement in research. Studies should consider screening participants for basic literacy and ability to complete mock sorting tasks or plan to have trained research staff available to assist when needed. Using cards with printed statements in the sorting phase may avoid computer literacy issues. Regardless of approach, study designs should report objective criteria for sort exclusions and exclusion rates in their results.

## Data Availability

All data generated or analyzed during this study are included in this published article. For additional details about the larger Voicing Outcomes Important to Care (VOICe) project, please see the referenced study.

## Abbreviations

DM: Diabetes mellitus
ED: Emergency Department
GCM: Group concept mapping
HbA1c, A1c: Hemoglobin A1c
IRB: Institutional Review Board
PAKSAB: Patient Advocate and Key Stakeholders Advisory Board
SD: Standard deviation
